# Comment on Leone et al. Association between Mediterranean Diet and Fatty Liver in Women with Overweight and Obesity. *Nutrients* 2022, *14*, 3771

**DOI:** 10.3390/nu15051139

**Published:** 2023-02-24

**Authors:** Elvira Alvarez Stehle

**Affiliations:** Dietetics and Nutrition Department, Robert Stempel College of Public Health & Social Work, Florida International University, Miami, FL 33199, USA; ealva217@fiu.edu

I read a recently published research study about the Mediterranean diet and its association with liver status with extraordinary interest. This research group focused on a population of overweight and obese women [1]. Although previous research had explained and provided an extensive guideline on the best screening methods for non-alcoholic fatty liver disease (NAFLD) detection, the study presently concerned went further and has helped explain how beneficial the implementation of NAFLD screening is for early liver disease detection [2,3]. Similarly, previous studies have shown the favorable effect of the Mediterranean diet on public health in line with other findings that consistently reflected the benefits of the Mediterranean diet in reducing cardiovascular disease and overall mortality [4,5,6]. Leone et al. did a great job targeting this large population and evaluating this diet and its impact on metabolism, particularly assessing the risk of fatty liver disease [1]. They provided evidence of the Mediterranean diet’s effectiveness in liver disease prevention [1].

However, it would be increasingly constructive for future research associating dieting and favorable health outcomes to use new sources for the MEDAS (14-item Mediterranean Diet Adherence Screener) validation tool other than the PREDIMED study [7]. Although PREDIMED was one of the most influential randomized trials ever, in June 2018, the trial was retracted and republished because of the detection of severe protocol deviations [8]. It might be helpful to reference another source for MEDAS validation, such as the study conducted by Garcia-Conesa et al., which provides reliable research and the ample corroboration of the 14-item Mediterranean Diet Adherence Screener [9].
